# Integrating expert knowledge in a GIS to optimize siting decisions for small-scale healthy food retail interventions

**DOI:** 10.1186/s12942-016-0048-6

**Published:** 2016-06-16

**Authors:** Richard Casey Sadler

**Affiliations:** Department of Family Medicine, Michigan State University, 200 E 1st St, Flint, MI 48502 USA

**Keywords:** Mobile markets, Food deserts, GIS, Expert knowledge, Multi-criteria decision making, Analytic hierarchy process, Public participatory geographic information systems, Community-based participatory research

## Abstract

**Background:**

The availability of healthy foods in a neighborhood remains a key determinant of diet and diet-related disease in disadvantaged communities. Innovative solutions to the ‘food desert’ problem include the deployment of mobile markets and healthy corner store initiatives. Such initiatives, however, do not always capitalize on the principles guiding retail development and the possibilities of GIS-based data. Simultaneously, community partners are not always engaged effectively in the planning for such interventions, which limits acceptability and suitability of such work.

**Methods:**

This paper highlights the results of a participatory mapping exercise to optimize the siting of a planned healthy food retail intervention in Flint, Michigan. Potential sites are chosen by engaging experts in a three-stage mapping process that includes the analytic hierarchy process and point allocation of five key variables (including food access, socioeconomic distress, population density, access to transit, and proximity to neighborhood centers), as well as direct mapping of suitable sites.

**Results:**

Results suggest a discrete set of areas—primarily in the northwestern quadrant of the city—where small-scale healthy food retail interventions might be most strategically located. Areas with the most consistent overlap between directly mapped sites and very high levels of suitability align well with neighborhoods which are distant from existing grocery stores.

**Conclusions:**

As a community-based strategy, this increases the opportunity for effectively improving neighborhood access to healthy foods by optimizing the potential sites for healthy food interventions. Community partners have already been active in using these results in project planning for just such an intervention.

## Background

Inadequate consumption of healthy foods such as fruits and vegetables remains a critical factor driving the nation’s health crises around obesity, diabetes, and other diet-related diseases [1, 2]. Research continues to show links between access to and consumption of healthy foods, as well as economic inequalities in access such that poorer neighborhoods have poorer access to healthy foods [3–5]. This compounding of social and physical inequalities highlights the troublesome deprivation amplification effect [6], highlighted by research showing that healthy food consumption declines when food security is threatened [7, 8]. Troublesome is that, in spite of programs such as SNAP and Double-Up Food Bucks which are intended to make foods more affordable, when residents lack geographic access to grocery stores and healthy food outlets, they simply cannot reach these stores to purchase healthy foods.

Considerable investment has been made recently in helping traditional food retail return to underserved urban areas [9, 10]. More recent work has advocated more flexible retail endeavors such as food trucks, mobile markets, or convenience store retrofitting due to the lower associated costs [11]. A frequent continuing problem of these place-based retail reinvestment decisions lies in a poor spatial conceptualization of risk factors for unhealthy food consumption; in many studies, existing knowledge on healthy food access, socioeconomic distress, and other determinants is not incorporated. Research is therefore needed to build on the evidence base for siting decisions around healthy food retail.

One method for optimizing land use siting decisions has been the use of multi-criteria decision making (MCDM). This practice facilitates the incorporation of diverse expert opinions into a coherent framework for suitability analysis of potential sites for investment [12, 13]. Despite its extensive use in land use planning and resource conservation, however, uptake has been slow within the food desert literature. Further, mapping exercises exhibiting too steep a learning curve may prevent effective participation by stakeholders [14]. The objective of this research is therefore to use local expert knowledge about social and physical factors that influence healthy food consumption to optimize potential locations for healthy food interventions in socioeconomically distressed neighborhoods in Flint, Michigan.

### Small-scale healthy food retail interventions

Initial interest in food retail interventions arose out of work on food deserts, premised on the idea that retail consolidation has had deleterious effects on the impoverished communities which were abandoned [15]. Considerable investment and research has been dedicated to tracking the impact of new grocery stores on diet and health, but the effects of these investments are not equivocal [16–19]. Researchers have cautioned that theoretical perspectives focused too heavily on individual behavior change—on which many retail interventions rely—may miscast the problem on the community [20–22], which can create hesitancy among future would-be investors.

In contrast, community-based approaches which engage local partners in planning and implementation of retail interventions may hold considerable opportunity [23]. Such approaches are more sensitive to the reality that ‘silver bullets’ do not exist for behavior change [24], and instead implicitly recognize the need for an alternative that recognizes the interplay between social structures and individual behavior [25]. Community-based work can serve as one such intermediary by increasing awareness of and promoting locations selling healthy food options, which can be critical to supporting adaptive strategies employed by residents living in food deserts [26, 27]. This pursuit of increasing visibility is particularly important because of the notion that residents in communities without supermarkets perceive fewer nutritious food options [27, 28].

Investments in existing corner stores and new mobile markets—hereafter referred to as small-scale healthy food retail (SSHFR) interventions—are twin ideas for addressing community nutrition needs on a smaller scale [29, 30]. Corner stores offer promise because many people routinely visit such stores [31], and they have connected with urban farms to increase retail-based fresh food access [32]. Elsewhere, new markets have been established in food deserts to connect low-income residents to healthy food options [33]. Mobile markets, meanwhile, can integrate local community supported agriculture with a more flexible distribution format that enhances the customer base [34]. Other studies have found increased fruit and vegetable consumption among mobile market shoppers [35], particularly when basic spatial analysis have been used to determine area-specific need [36]. More sophisticated spatial analyses incorporating both low access and low income variables, meanwhile increase the certainty and validity of predictive models for optimal site selection [37].

### Expert knowledge and multi-criteria decision making

These results suggest that GIS-based methods can be effective at optimizing SSHFR interventions. But to date, expert knowledge of social and physical factors has not been incorporated into the planning for such interventions. Within the land use planning literature, suitability modeling is commonly used to determine optimal sites for development or conservation [38, 39]. Multiple attributes (often defined as categorical variables) are combined through map algebra to suggest the best sites for use, with higher scores indicating greater suitability. Because unweighted sums lack sophistication in important land use planning processes, multi-criteria decision making (MCDM) is used to account for the reality that some variables are more important than others [40].

Variable weights also cannot be arbitrarily assigned; thus, expert knowledge from key stakeholders is a common evidence-based method for assigning weights to variables [41, 42]. Because this weighting so strongly impacts the final results [43], the identification of key community stakeholders and the use of rigorous analytical methods are important for obtaining the best results. Experts have been engaged in the past to not only derive effective results, but also lend expertise to problem identification and study design, especially when they possess local knowledge that content experts may lack [44]. In that study, local stakeholders were seen as a critical piece of the decision-making process, as their involvement increased the sense of local ownership of the work.

Regarding rigorous methods, the analytic hierarchy process (AHP) is effective at integrating expert opinion into variable weight assignment because of its simple pairwise comparison approach [45]. Experts using AHP simply pit variables against one another in a ‘round-robin’ format, assigning importance scores to the ‘winning’ variables on a scale of 1–9 (Table 1).

**Table 1:** Scale used for pairwise comparisons in the analytic hierarchy process

Intensity of importance	Definition
1	Equal importance
3	Moderately more important
5	Strongly more important
7	Very strongly more important
9	Extremely more important
2, 4, 6, 8	Intermediate values

Pursuant to weight assignment by experts, a reciprocal matrix (*A*) is constructed:

$$A = \left[ {\begin{array}{*{20}c} {a11} & \cdots & {a1n} \\ \vdots & \ddots & \vdots \\ {an1} & \cdots & {ann} \\ \end{array} } \right]$$

In this table, a_ij_ represents the head-to-head comparison of attribute *i* and attribute *j*, while *n* represents the total number of attributes. A priority vector is calculated from a normalization of the matrix to derive an eigenvector in which the final weights reside. The final weights assigned by each expert can then be averaged to derive a final composite score [46]. Because of the potential for human error in weight assignment, a weighted sum vector can also be calculated to derive a maximum eigenvalue and assign an internal consistency score to each expert’s weights. The calculation for a consistency vector involves a sum of the products of the weighted sum vector and the priority vector (*w*_*i*_), and is given as:

$$\lambda \hbox{max} = \mathop \sum \limits_{i} \mathop \sum \limits_{j} aij*wi$$

Using the value for λmax, a consistency index (*CI*) is calculated as:

$$CI = \frac{{\lambda { \hbox{max} } - {\text{n}}}}{n - 1}$$

To determine whether the expert’s weights are internally consistent, a consistency ratio (*CR*) is computed by dividing *CI* by a predefined random consistency index (*RI*) which varies by the size of the n × n matrix^1^ [47]. When the *CR* is less than 0.1, the individual expert’s weights are considered internally consistent. Functionally, this means that when inconsistencies arise, the researcher can revisit the weights with the expert to clarify unequal weighting.

An example of this process (shown in Table 2) assumes that socioeconomic distress is the most important variable, garnering 46 % of the total weighting (all weights are underlined in the eigenvector of the priority vector), with availability (22 %), density (13 %), centers (12 %), and bus stops (7 %) receiving less of the total. These weights are derived from the AHP weights table scored by the hypothetical expert: the high scores in the socioeconomic distress row generate the stronger weight for that variable in the priority vector. As noted above, the weighted sum vector is used to generate a consistency vector. The *CR* score of 0.01 indicates that this vector is internally consistent. This process is applied similarly regardless of the variables used, as the task of the expert is simply to apply comparative weights to the variables under consideration.

**Table 2:** Example of AHP weighting exercise

	A	S	D	B	C	Eigen vector
AHP weights table						
Availability	1.00	0.33	2.00	3.00	2.00	
Socioeconomic distress	3.00	1.00	3.00	5.00	4.00	
Density	0.50	0.33	1.00	2.00	1.00	
Bus stops	0.33	0.20	0.50	1.00	0.50	
Centers	0.50	0.25	1.00	2.00	1.00	
Priority vector (Eigenvector is suitability score)						
Availability	0.19	0.16	0.27	0.23	0.24	0.22
Socioeconomic distress	0.56	0.47	0.40	0.38	0.47	0.46
Density	0.09	0.16	0.13	0.15	0.12	0.13
Bus stops	0.06	0.09	0.07	0.08	0.06	0.07
Centers	0.09	0.12	0.13	0.15	0.12	0.12
Weighted sum vector						
Availability	0.22	0.15	0.26	0.22	0.25	1.09
Socioeconomic distress	0.65	0.46	0.39	0.36	0.49	2.35
Density	0.11	0.15	0.13	0.14	0.12	0.66
Bus stops	0.07	0.09	0.07	0.07	0.06	0.36
Centers	0.11	0.11	0.13	0.14	0.12	0.62
Consistency vector						
Availability	5.07		n	5		
Socioeconomic distress	5.13		λ	5.06		
Density	5.02		CI	0.02		
Bus stops	5.04		RI	1.12		
Centers	5.03		CR	0.01		

Because of the need for evidence-based metrics to define optimal sites for SSHFR interventions, for this paper AHP is incorporated into a multi-stage expert opinion participatory GIS exercise. This approach provides an analytical method for incorporating community knowledge to inform spatial decision-making in an impending SSHFR intervention.

### Study area

This approach is particularly valuable in Flint, Michigan, because a long history of retail disinvestment and a more recent public health emergency around lead in drinking water have spurred increasingly urgent discussion about SSHFR options to address nutritional needs [48]. Flint’s food retail network is at an advanced stage of consolidation. Beginning in the late 1960s with the passage of a Fair Housing Ordinance, and accelerating with severe deindustrialization due to layoffs by the city’s former primary employer—General Motors—white and later middle class flight has cut the city population in half [49–52]. Since 2012, the Flint urban area has lost six grocery stores, including five chains. Even so, many organizations are active in alternative strategies to provide nutritious foods, including through urban gardening, farmers’ markets, nutrition education, and expansion of food assistance programs [53, 54]. The approach taken in this paper is intended to provide an evidence base for these and other community organizations to build upon in planning for SSHFR interventions.

## Methods

The methods for this research include: (1) mapping key social and physical factors affecting healthy food consumption; (2) identifying and engaging key community partners in a three-stage mapping process to gauge expert knowledge on the issue; and (3) combining the weighted maps to determine the most consistent and suitable sites for locating SSHFR interventions.

### Preliminary mapping

**Fig. 1:**
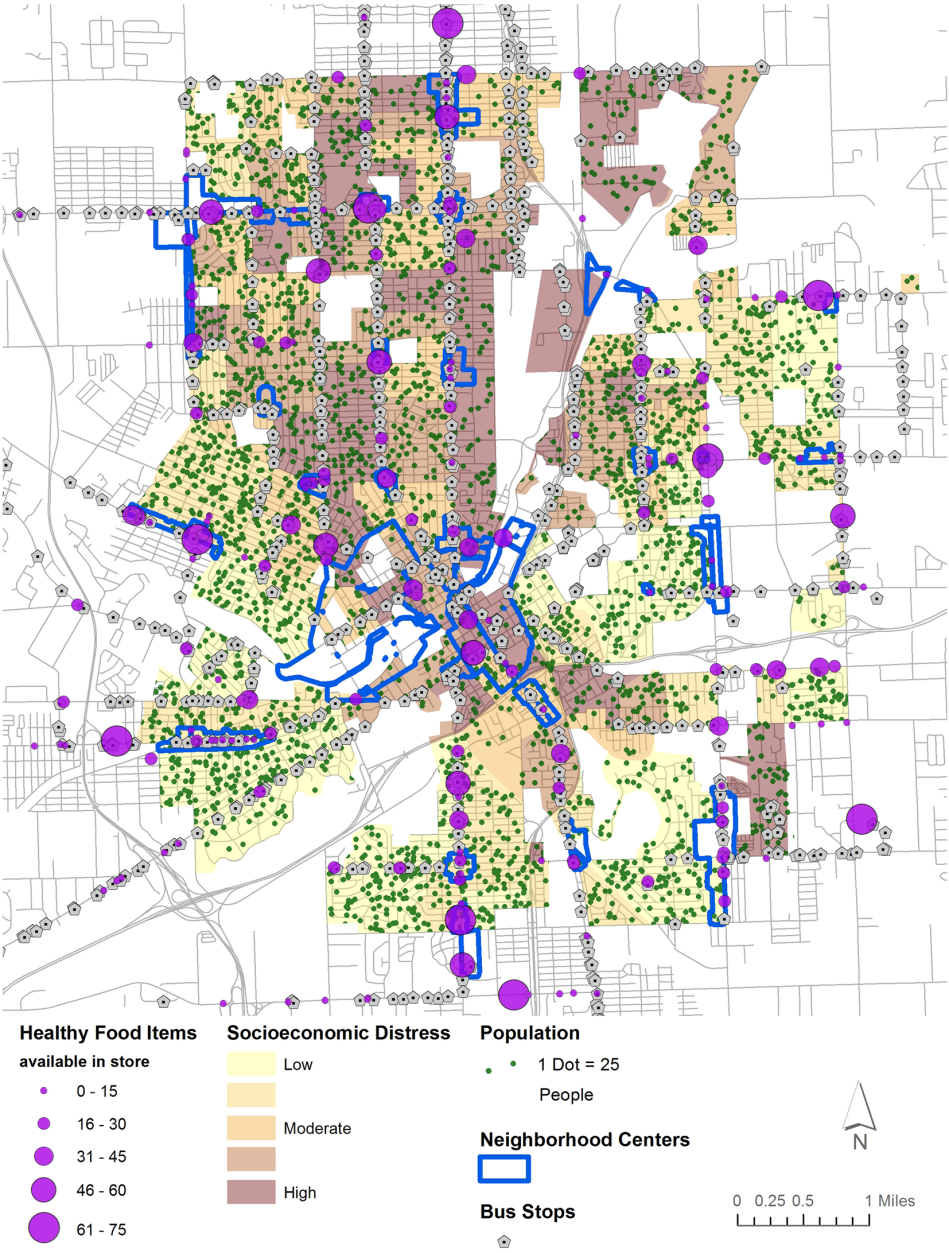
Variables used in multi-criteria analysis

Based on community work by the author on issues of healthy food access, and in partnership with participants from Edible Flint—Flint’s local food collaborative and advocate for land use-oriented interventions to facilitate healthy eating—five variables were chosen for consideration in planning for SSHFR interventions: healthy food availability, socioeconomic distress, population density, bus stops, and neighborhood centers (mapped in Fig. 1).

Generally, the purpose is to highlight areas with *poor* healthy food availability, *high* socioeconomic distress, *high* population density, *close* proximity to bus stops, and a presence *within or near* designated neighborhood centers. Areas with these corresponding characteristics, therefore, would be most suitable for future SSHFR interventions. After initial mapping and spatial analysis, each layer was converted to a categorical variable and assigned a value ranging from 0 to 10 to facilitate map algebra (shown in Table 3); thus 10 is the highest hypothetical value that any location could receive. While fuzzy classifications have previously been used alongside expert knowledge weighting [55–57], categorical variable construction remains a common element of expert knowledge. Part of its utility lies in the simplicity of boundary definitions, which allow experts to shape their responses based on these classes. Further, discrete barriers between classes enable greater simplicity in deriving suitability for planning purposes.

**Table 3:** Summary of variables used in survey

Variable	Score	Category
Healthy food availability	10	Worst availability
	8	2nd worst availability
	5	Average availability
	2	2nd best availability
	0	Best availability
Socioeconomic distress	10	Most distressed quintile
	8	2nd most distressed quintile
	5	Average distress quintile
	2	2nd least distressed quintile
	0	Least distressed quintile
Population density	10	Densest quintile
	8	2nd densest quintile
	5	Middle quintile
	2	2nd least dense quintile
	0	Least dense quintile
Proximity to bus stops	10	1/8 mile
	8	1/4 mile
	5	3/8 mile
	2	1/2 mile
	0	>1/2 mile
Neighborhood centers	10	In center
	5	Within 1/4 mile
	0	>1/4 mile

*Healthy food availability* was derived from a food store assessment conducted in the summer of 2012 [58] and updated based on recent store closures verified through site visits. The original food store assessment was conducted at 161 stores in or within 2 miles of the City of Flint by a team of 3 evaluators [58]. For this paper, scores were extrapolated to an additional 117 stores based on size, type, and location of the stores. For example, stores within a chain or a similar store that were not evaluated were given the average score of the evaluated stores from that chain or type. Store size was validated by site visits and through retail listings. Doing so allows for a comprehensive estimate of the amount of healthy food available throughout the community, based on a sample of 58 % of the total.

**Fig. 2:**
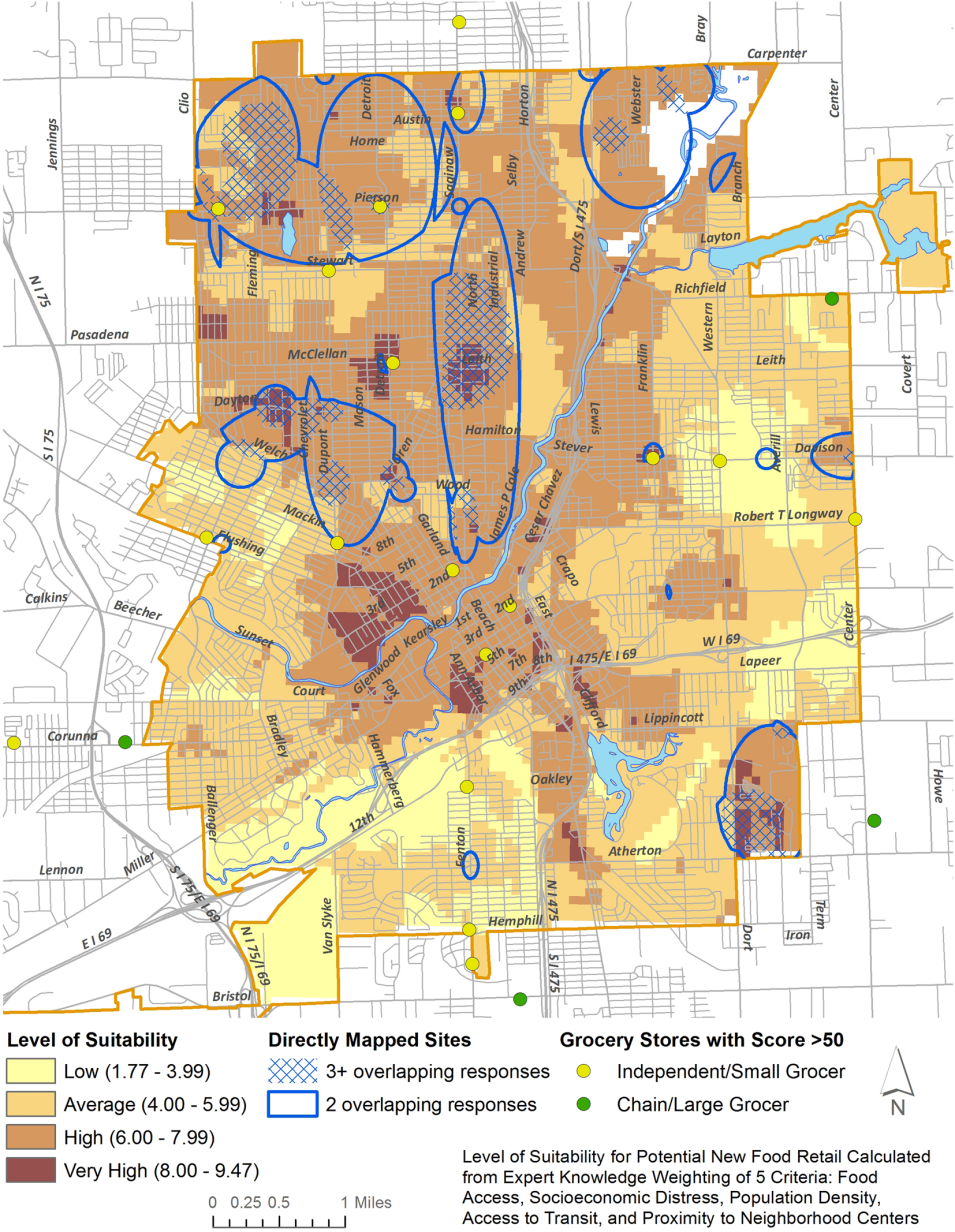
Composite suitability map of AHP and PA with overlapping directly mapped sites

The assessments included 62 specific healthy food items (including dairy, meats, and grains), as well as open-ended questions on a range of fresh, frozen, and canned fruits, vegetables, and legumes. From these assessments, each store was assigned a healthy food score sum ranging from 0 to 75 in the Flint community; the highest possible score a store could receive was 125 (based on the total number of unique items available). For visualization purposes in Fig. 2, a score of at least 50 was considered adequate to represent a small grocery store (based on common characteristics of these stores. A kernel density surface was calculated (as in past research) to show the relative availability of healthy foods in the study area [59]. Finally, the surface of availability was converted to five categories by equal interval—a classification scheme shown to perform better than others (e.g. quantiles) when considering a narrow range of values [60]. Areas with poorer food availability scores thus received higher suitability scores (Table 3); the use of equal interval categories meant a clearer representation of ‘poor access’ areas than a quantile classification would have provided. In particular, this dataset builds on previous contributions such as Widener et al.’s [37, 61] because a comprehensive view of healthy food availability can be integrated into the spatial model.

*Socioeconomic distress* was derived from four census block group (CBG) level variables [62] which together serve as a proxy for material and social deprivation. An unweighted z-score sum was computed from rates of low educational attainment, unemployment, poverty, and lone parenthood (as created in Pampalon et al., and used for this study area in Sadler et al.) [63, 64]. The z-score sums were divided into five quantiles and scores were assigned based on the category of socioeconomic distress, with more distressed CBGs receiving higher suitability scores under the presumption that disadvantaged neighborhoods required more attention from SSHFR interventions.

*Population density* was derived by calculating the number of people per square mile within each CBG. Thresholds were chosen by dividing the CBGs into quintiles. CBGs with higher population densities received higher suitability scores. This assignment reflects the need for a SSHFR to serve as many people as possible to be successful financial and socially.

*Proximity to bus stops* was calculated by running network analysis in increments of 1/8 mile (1/8, 1/4, 3/8, 1/2), reflecting commonly used thresholds for walking zones around bus stops^2^ [65, 66]. Zones closer to bus stops were given higher suitability scores, under the assumption that patrons visiting new SSHFR would want to economize on distance travelled.

*Presence within or near designated neighborhood centers* addresses the city’s new master plan, which specifically enumerates key zones for commercial uses [67]. While food trucks/mobile markets are allowed by right in many residential zones, other food retail uses are allowed only within commercial zones. Some variances might be granted on properties near commercial zones, so the classification for this categorical variable reflects this possibility (Table 2). Neighborhood centers are also located at visible focal points in the community; thus, a SSHFR would generally be more likely to succeed here.

### Engaging community partners

With the initial mapping layers computed, the next step involved engaging key community partners with expert knowledge on food access issues. The motivation behind engaging these partners comes from two related but often unconnected perspectives: public participatory GIS (PPGIS) and community-based participatory research (CBPR). PPGIS was popularized as such in 1996 as the growth of computer-based mapping systems lowered the barriers to such work, and from the need to integrate public and expert involvement in spatial decision-making processes [68]. CBPR has a longer history—growing from Lewin’s mid-20th century participatory action research—and aims to address social inequalities and achieve social change by combining community knowledge with action [69–71]. Despite their co-existence over the past 20 years, very little work has explicitly approached the idea of reducing health inequalities by using PPGIS with a CBPR approach, with the earliest work occurring in the last decade [72] and fewer than two dozen articles written on the nexus overall. In a related vein, work on volunteered geographic information (VGI) and the ‘wikification of GIS’ has likewise proliferated in recent years, as the public is increasingly able to contribute to mapping exercises [73–75]. The distinction between CBPR and VGI or wikified GIS may seem semantic, but explicitly adopting CBPR as the theoretical framework carries with it a clearer connotation to community-engaged and community-led research. In contrast, VGI and wikified GIS do not explicitly require community engagement at all stages of a project. Even so, the overlaps in methodology and theoretical orientation offer an opportunity for deeper inquiry in the future.

Combining PPGIS with CBPR means that community partners were engaged at all stages of the process, including tool design, expert input, and interpretation of results. In consultation with community partner Edible Flint, a list of 11 experts was compiled from anti-hunger and local food organizations, as well as local foundation contacts with some stake in healthy eating initiatives. All partners are motivated by the same basic goal, and are already active in efforts to bring SSHFR or programs to underserved neighborhoods. Given the focus of this work on ameliorating community food insecurity, these partners represented the breadth of experts on this subject within the community, and are thus well-suited to this exercise. Experts were contacted with a request to participate in a three-stage participatory mapping exercise, and the author met with each individually. The structure of this exercise was partially derived from earlier research on natural resource conservation planning [38].

In the first stage, experts evaluated the relative importance of the five variables directly against one another using the AHP method. Experts chose between each variable, and assigned a score indicating how much more important one variable was versus another. The second stage involved a point allocation (PA), whereby the experts doled out 100 total points to the 5 variables. This provided the experts with a second look at the variables overall and enabled an alternative method of assigning weights. Given that many experts also had keen knowledge of specific communities, direct mapping constituted the third component of the exercise. Experts were invited to mark anywhere they felt SSHFR would be of value. Experts did not see the results of the AHP/PA exercise prior to the direct mapping; thus, their opinions on this exercise were not influenced by the results of the AHP/PA. Throughout the process, experts were encouraged to ask clarifying questions and make any additional comments, especially regarding how to improve the strategy for optimizing locations for SSHFR.

### Combining data

Once data collection was completed, AHP results were inserted into individual matrices to derive weights and ensure the *CR* was below the upper threshold of 0.1. AHP results were then compared to the PA results to determine cross-method consistency within variables and among experts. Using the values from the preliminary mapping (Table 2), the AHP and PA weights for each variable were assigned to generate suitability layers. A third suitability layer was constructed by digitizing and overlaying each expert’s direct mapping results. This process enabled the determination of the most suitable and consistent sites for locating new SSHFR interventions.

## Results and discussion

Suitability maps were derived for each of the three data collection methods: AHP, PA, and direct mapping. Both the AHP and PA maps were derived from the average of the weights assigned by the experts (Table 1), for AHP as in Table 2 and for PA through directly assigning points totaling to 100. For AHP, socioeconomic distress (25.8 %) and availability (25.5 %) received nearly the same weight assignment—suggesting nearly equal importance—followed by centers (20.6 %), density (15.8 %), and bus stops (12.8 %). The PA scores were slightly less balanced, with availability scoring highest (31.4 %), followed by socioeconomic distress (23.0 %), density (18.8 %), centers (15.5 %), and bus stops (11.4 %).

Initially, 5 of 11 experts had AHP consistency indices above the allowable limit. Follow-up discussion addressed these inconsistencies for the calculation of final, internally consistent scores combined into a composite score. Thus the final weighting used for the mapping (in Table 4) gave availability the highest weight (28.4 %), followed by socioeconomic distress (24.4 %), centers (18.0 %), density (17.3 %), and bus stops (11.8 %). As a final check of consistency, Table 4 also highlights the two-tailed paired *t* test—p-values for individuals’ AHP and PA scores. These p-values represent the probability that the two groups are different purely by chance, thus low scores (p < 0.05) represent significant difference. High scores therefore signify that the AHP-derived weights and the PA-derived weights are not significantly different from one another.

**Table 4:** Average of expert assigned weights for AHP and PA, and paired t test results

Variable	AHP	PA	Composite weight	Significance (p value of t test)
Availability	0.255	0.314	0.284	0.222
Socioeconomic distress	0.258	0.230	0.244	0.305
Density	0.158	0.188	0.173	0.281
Bus stops	0.123	0.114	0.118	0.592
Centers	0.206	0.155	0.180	0.321

Given the lack of a significant difference between AHP and PA and for the sake of brevity, a composite average of the two was created (shown in Table 4) to derive Fig. 2. Each location received a suitability score between 0 and 10, generated by multiplying each variable score from Table 3 with the corresponding composite weight from Table 4. To further highlight the close relationship between the AHP and PA maps, a difference map showing the percent error is included as “Appendix”. This “Appendix” is used as a guide to interpret suitable sites that received consistent scores between methods (error < 5 %).

**Fig. 3:**
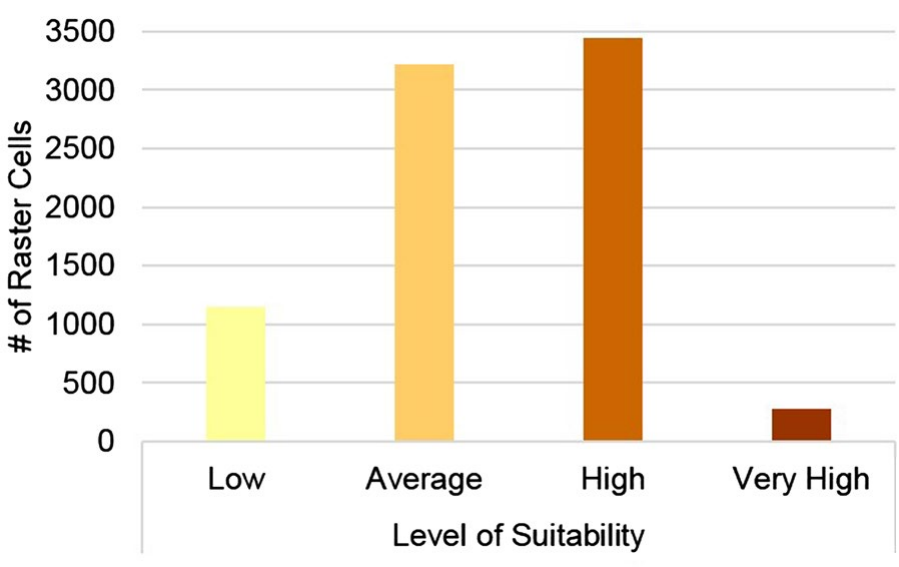
Distribution of raster cells from Fig. 2 in each suitability category

Figure 2 shows the results spatially, with scores ranging from 1.76 to 9.47. Suitability ‘zones’ were created to bin areas into ‘low’ (less than 4.00), ‘average’ (between 4.00 and 5.99), ‘high’ (between 6.00 and 7.99), and ‘very high’ (8.00 and up) suitability scores. These bins were chosen based on an assumed normal distribution of scores around a mean value of 5.00. The ‘very high’ suitability scores thus represent areas that captured 80 % of the total possible score, and which are at the confluence of poor food availability, high socioeconomic distress, high population density, close proximity to bus stops, and close proximity to neighborhood centers. Conversely, ‘low’ suitability sites scored poorly on most metrics, receiving less than 40 % of the total possible score. The relatively low distribution of sites with very high suitability (shown in Fig. 3) is a natural indicator of the utility of the weighting process for decision-making around SSHFR, as only the most suitable sites yielded high scores, preserving a broadly bell-curved distribution.

Spatially, the highest scores were found primarily in older neighborhoods immediately north and west of the city center. Additional areas were found in the far southeast and far north. Each of these areas fall in zones where the percent error is less than 5 %, and thus are considered consistent between weight assignment methods. The implication is that these neighborhoods have very high suitability by both the AHP and PA methods.

Further supporting their inclusion as ideal sites for SSHFR is that the areas align with sites circled in the direct mapping exercise. The blue outlines in Fig. 2 correspond to areas where at least 2 experts overlapped in their choices for ideal SSHFR sites, while the hashed areas signify places where 3 or more experts overlapped. Of note is the overlap of sites with very high suitability derived from AHP/PA with zones where 3 or more experts directly mapped as suitable. These include areas in the far northwest near Pierson Rd & Fleming Rd (all cited road names listed on Fig. 2); in the near north/west at Leith St & Saginaw St, Garland St & Oren Ave, and Dayton St & Chevrolet Ave; and in the far southeast at Atherton Rd & Dort Hwy. In every case, these intersecting very high suitability and directly mapped sites are also distant from existing grocery stores (shown as circles in Fig. 2). Additionally, each of these areas coincides with a community anchor, including churches, non-profits, community centers, and schools.

Two additional very high suitability sites immediately west of downtown are the current focus of considerable reinvestment, as the city’s downtown core has been redeveloping over the past 10 years as a result of growth in healthcare and education. While these did not overlap with directly mapped sites, they may hold additional opportunity as SSHFR sites because of the newly expanding developments in the immediate vicinity. Equally important, however, is that these neighborhoods still very much have food access needs. The gap between direct mapping and AHP/PA suggests that these methods thus have the capability to uncover sites that may be valuable as SSHFR intervention sites, despite not being considered intuitively suitable by community partners whose focus may be on other neighborhoods.

## Conclusions

The rationale for SSHFR has arisen from deficiencies in the conventional food system. In Flint, the problem is not a lack of food: it is an overconcentration of unhealthy food and a failure of traditional food retailers to serve inner-urban neighborhoods. The community-generated idea of SSHFR has recently gained traction from nutrition advocates as concerns over lead exposure remain high in light of the public health crisis caused by the deterioration of pipes leaching lead into the water system in Flint [48]. The timeliness and importance of a community-informed optimization for potential SSHFR sites in the Flint community cannot therefore be overstated.

The sites with very high suitability for a SSHFR reflected the experts’ emphasis on poor healthy food availability and high socioeconomic distress as the two key factors, but were also further focused on highly visible locations in neighborhood centers. The ability to take expert knowledge and convert it into a usable guide for planning a SSHFR intervention is of great importance, as financial constraints mean that residents in these neighborhoods are most likely to be relegated to shopping near home.

This PPGIS exercise had the effect of creating more connections among groups interested in SSHFR as an option for addressing inequalities in access to healthy foods. As well, the mapping results have already been used by several community groups in moving forward with SSHFR proposals, including a collaboration of community gardens advocates, operators of produce carts, farmers’ markets, agencies coordinating food distribution, non-profit/faith-based organizations, public health/healthcare institutions, and local/state government officials (most of whom were participants in this study). Input continues to be sought from the community on potential modification of the GIS model parameters, but most dialogue to date has been centered on using the research findings to deploy a SSHFR. The community continues to undertake assessments of readiness and funding opportunities to support SSHFR projects, and secured funding for a pilot project for the summer of 2016.

Thus, the community engagement conducted throughout this paper served as the first step toward building community buy-in of a SSHFR intervention, and in helping the community identify issues that were of concern to them in effectively deploying a SSHFR. This exercise helped build ownership of the idea of a SSHFR, enabled credit to be given where credit is due in the planning process, and served as a piece of the puzzle in breaking the cycle of research that is done *on* the community rather than *alongside* the community.

Beyond the Flint community, this work holds opportunity for framing SSHFR interventions in many other communities—particularly those like Flint who are struggling with declining populations and abandonment by traditional retail. Other researchers can replicate these methods to help optimize siting of facilities in their communities. The importance of this is rooted in the need to counter the conventional food system’s inclination to alienate already-marginalized populations. Although the current study cannot assert the effectiveness of the future SSHFR intervention, the assumption is that by engaging the community to choose optimal sites through a concerted CBPR approach, such efforts will be more likely to succeed. This work is therefore a template for best practices in siting SSHFR and integrating PPGIS with CBPR. Future work will continue to utilize the GIS approach by measuring the geographic reach and neighborhood-specific sales of SSHFR projects. Specifically, a follow-up study will measure the rate of sales and redemption of food assistance benefits stratified by suitability zone and neighborhood characteristics. Should the ‘high suitability’ zones return the highest sales and rates of redemption, the method would be validated as useful for deploying SSHFR interventions.

Ultimately, this work is intended to address the shortcomings of other food desert interventions by circumventing the frequent reliance on conventional food retail and by creating a data-driven approach to siting SSHFR which optimizes exposure to the greatest number of underserved residents. Subsequent programs can use this approach to improve the reach of such markets in food deserts.

## Figures and Tables

**Fig. 4:**
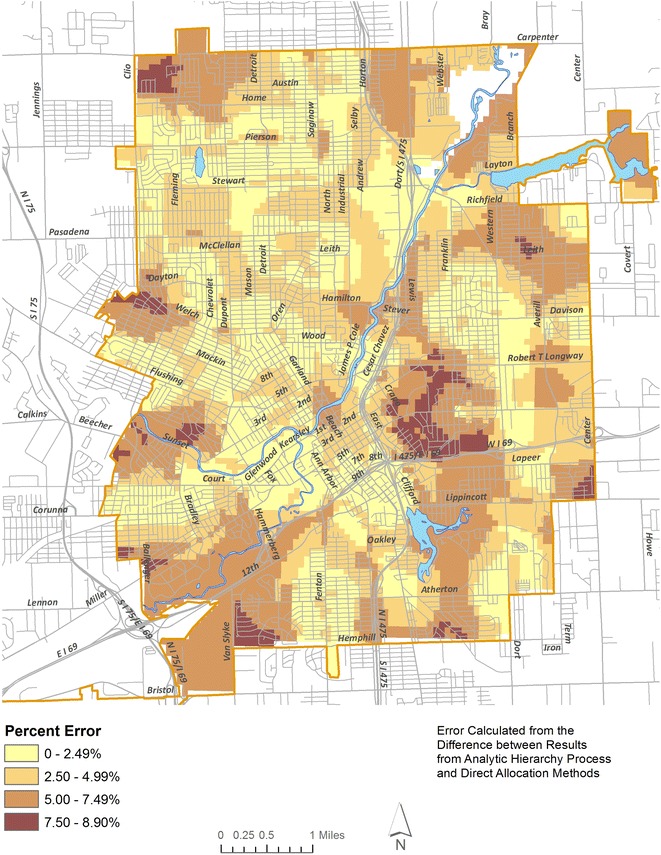
Difference map showing error between AHP and direct allocation methods

## Appendix

See Fig. 4.

## Abbreviations

AHP: analytic hierarchy process
CBG: census block groups
CBPR: community-based participatory research
CI: consistency index
CR: consistency ratio
MCDM: multi-criteria decision making
PA: point allocation
PPGIS: public participatory GIS
RI: random consistency index
SSHFR: small-scale healthy food retail

## Footnotes

1 In the current study, the random consistency index is 1.12, based on n = 5 variables.

2 For example, Neilson and Fowler [65] found 90 % of all transit riders travelled less than 1/2 mile from their point of departure, while transit use for destinations within 1/8 mile was 3 times greater than for 1/4 mile. Untermann [66] likewise used 1/4 mile as a walking threshold.
